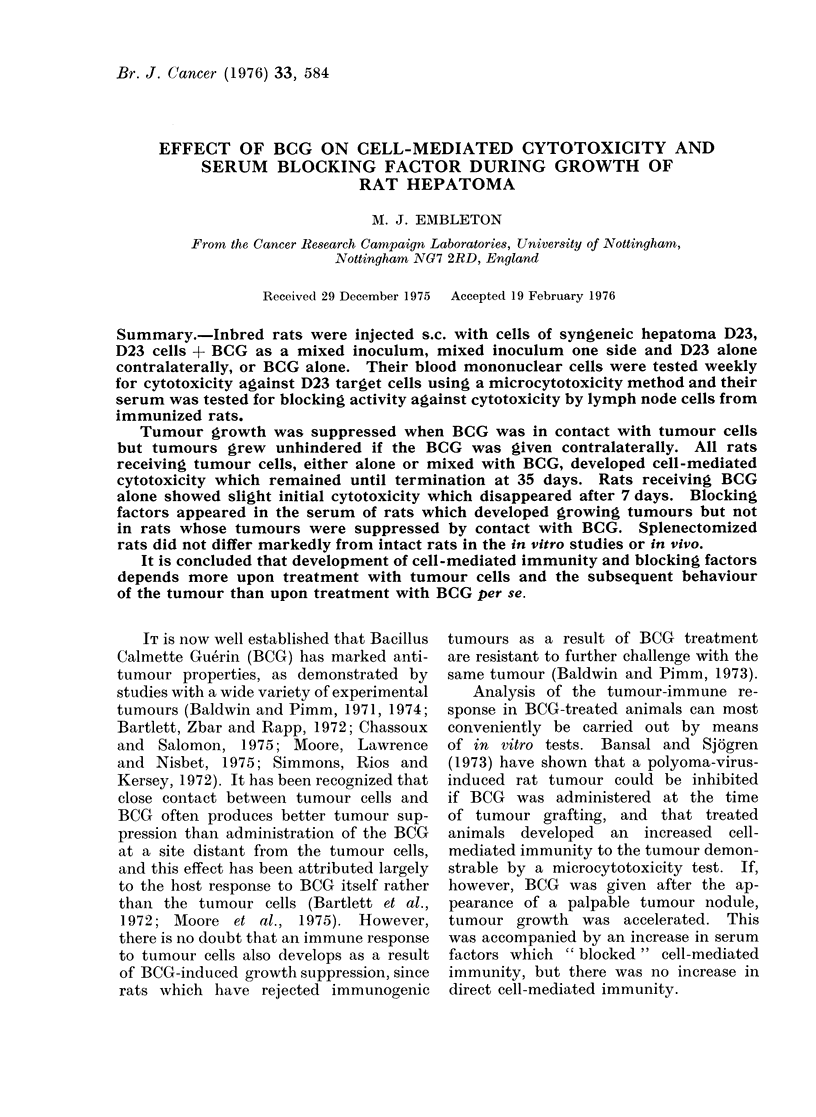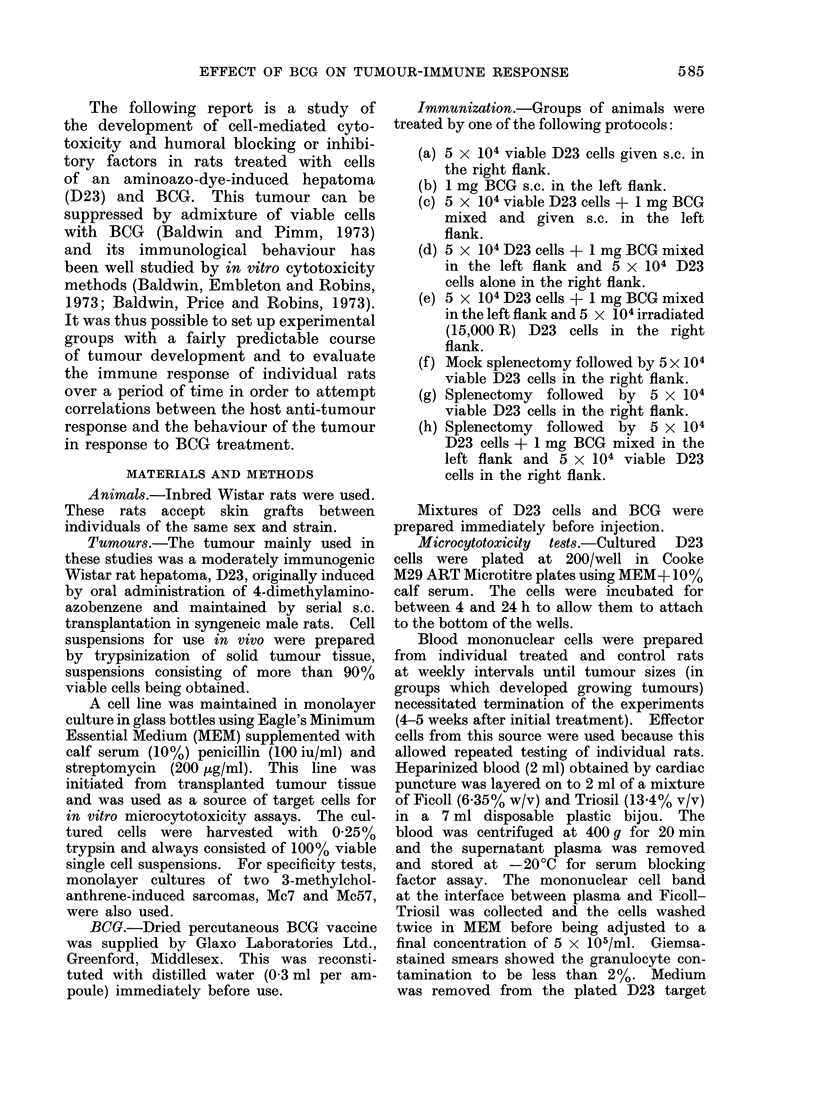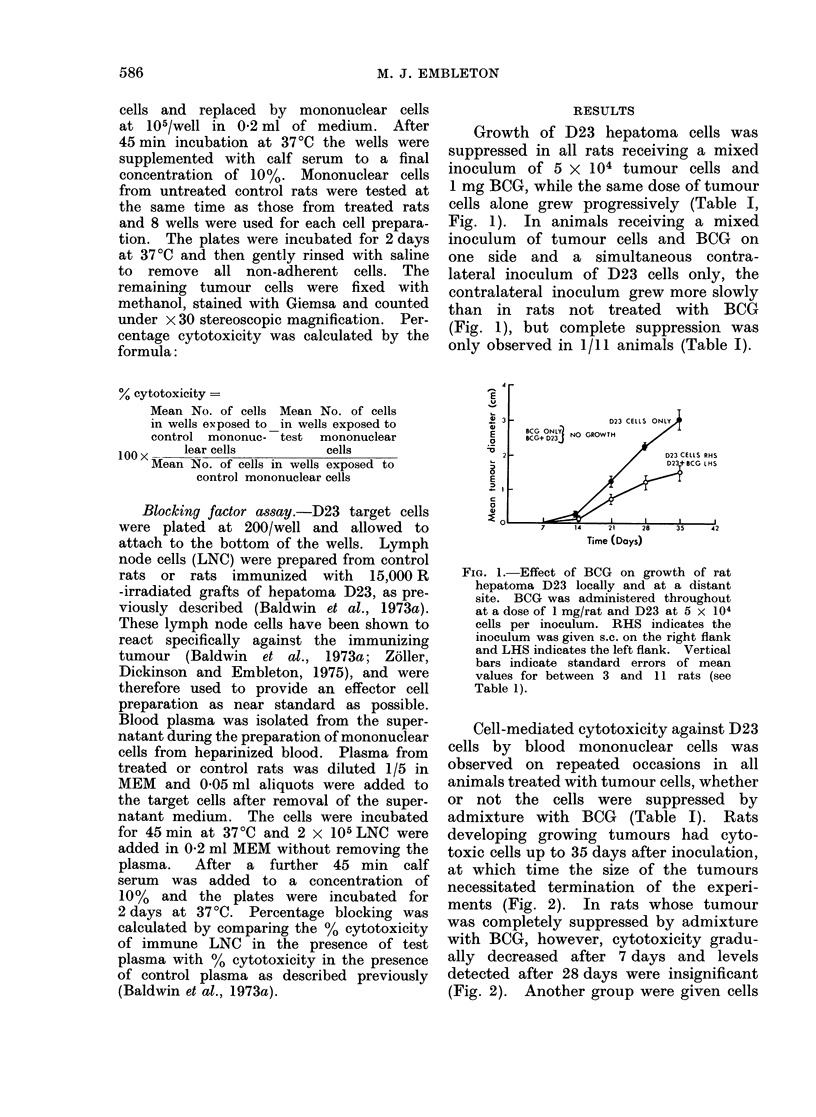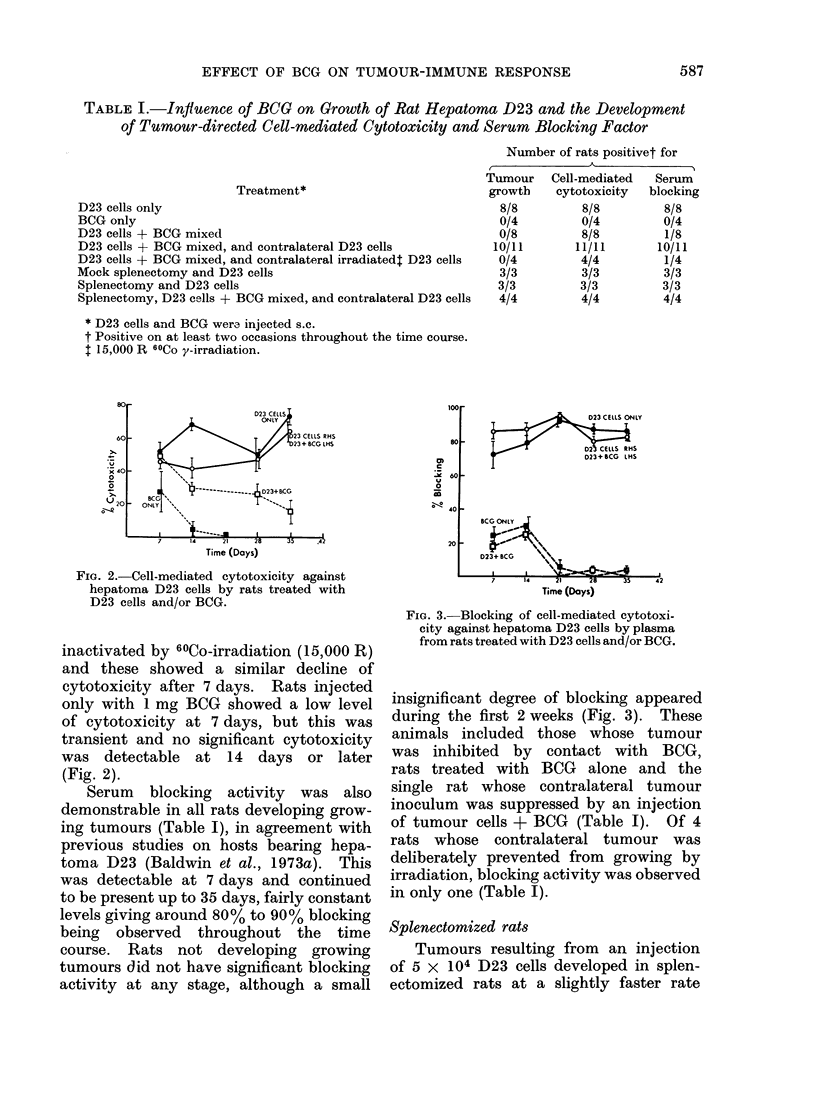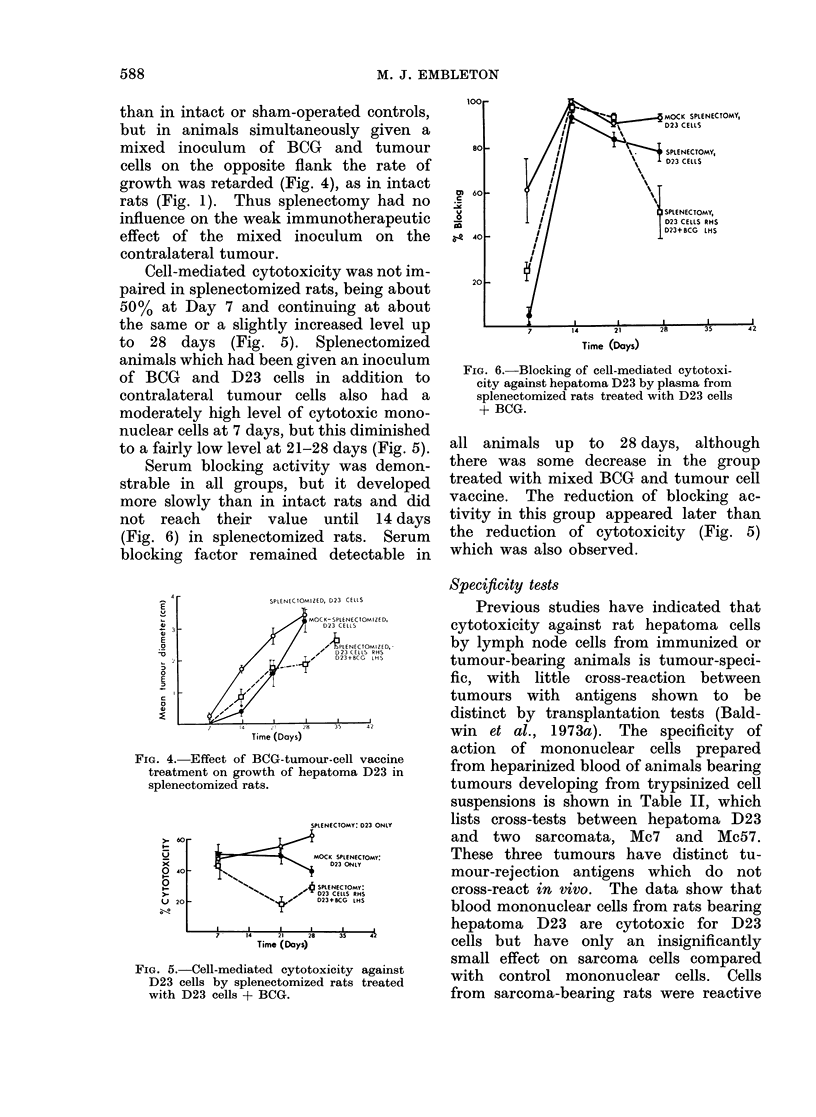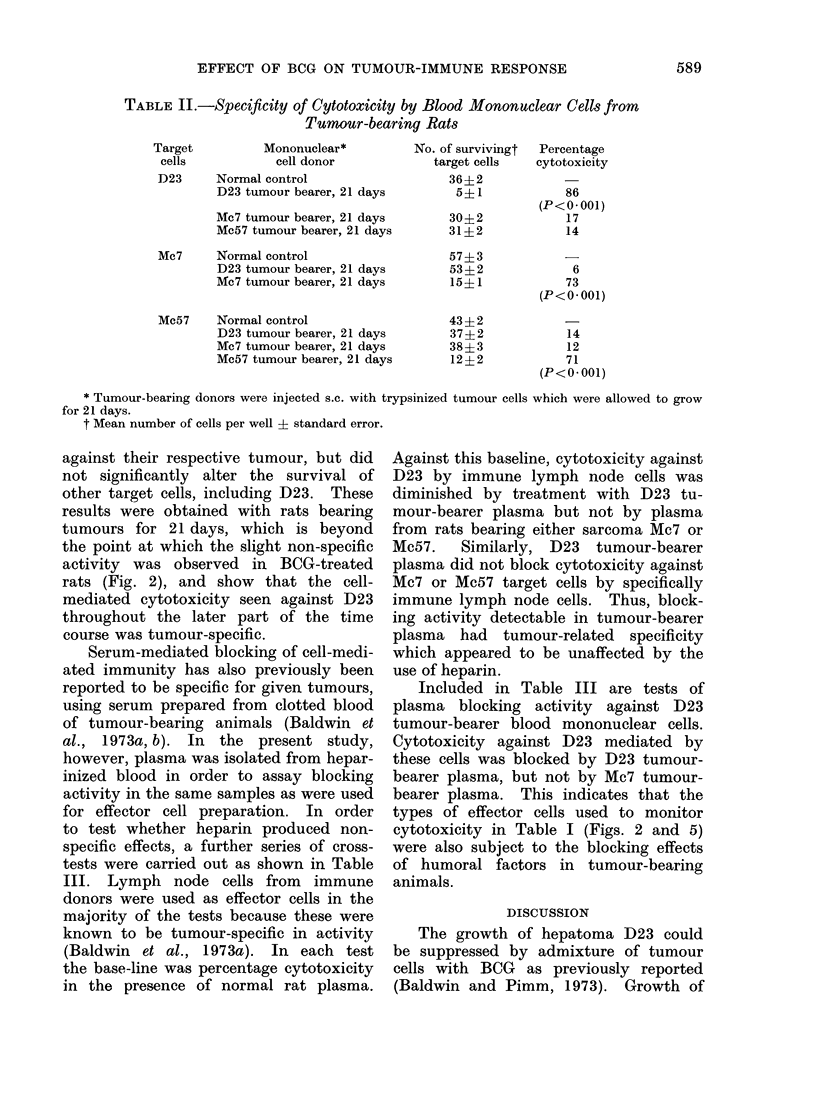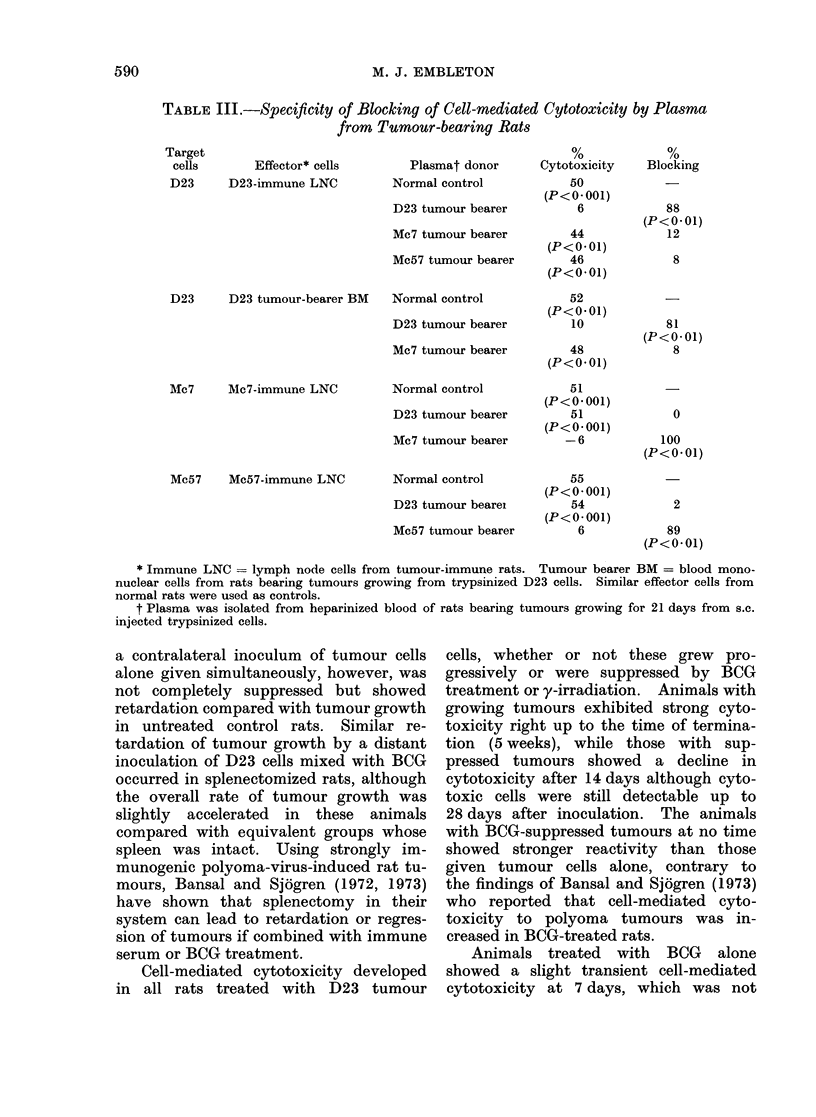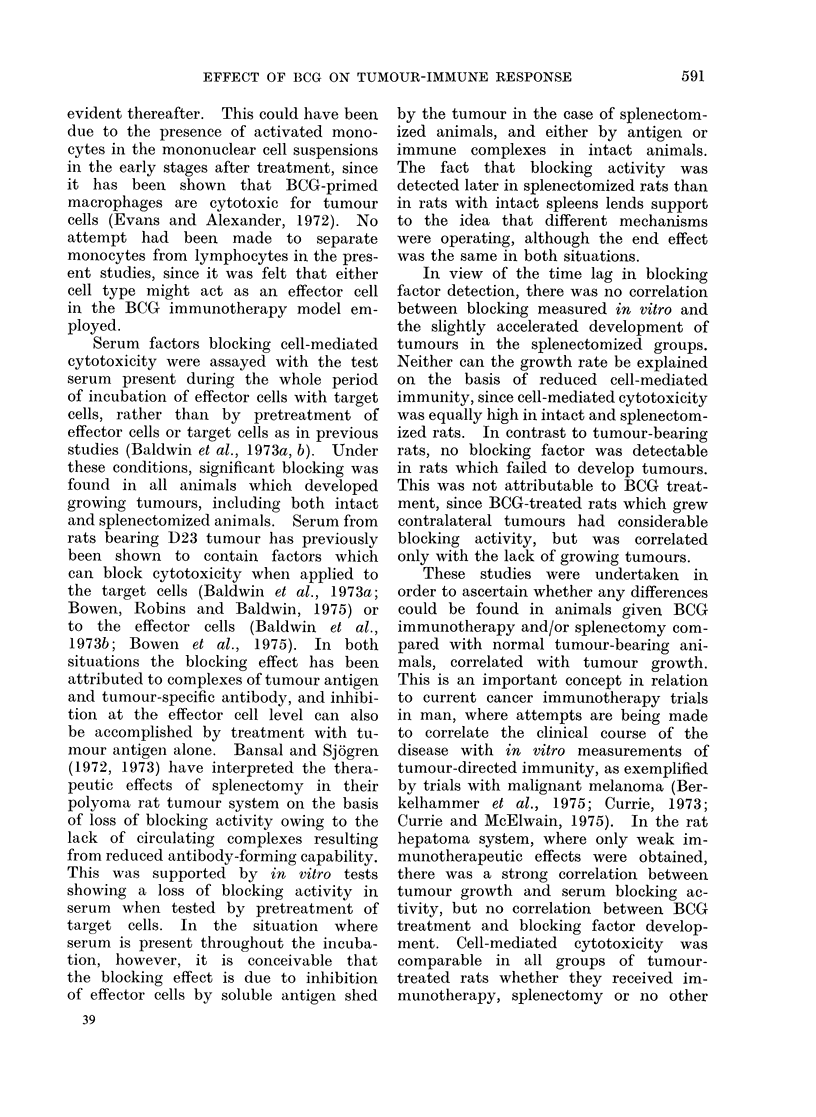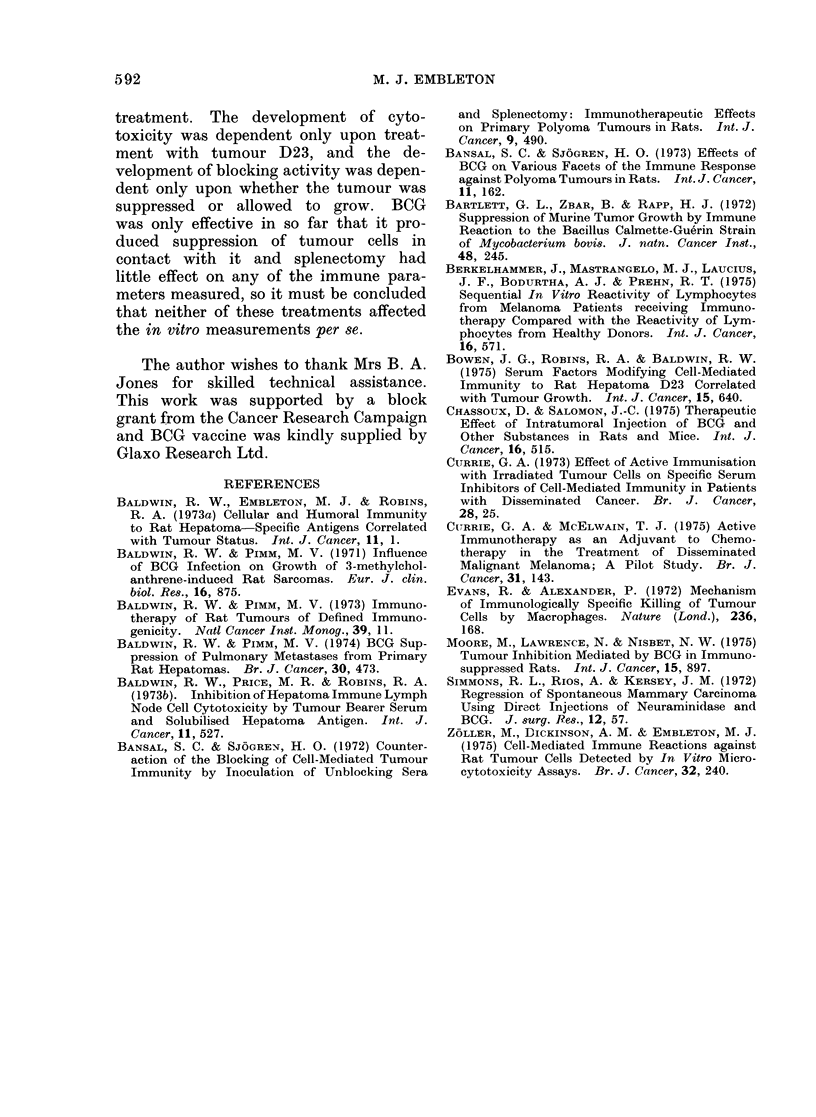# Effect of BCG on cell-mediated cytotoxicity and serum blocking factor during growth of rat hepatoma.

**DOI:** 10.1038/bjc.1976.95

**Published:** 1976-06

**Authors:** M. J. Embleton

## Abstract

Inbred rats were injected s.c. with cells of syngeneic hepatoma D23, D23 cells + BCG as a mixed inoculum, mixed inoculum one side and D23 alone contralaterally, or BCG alone. Their blood mononuclear cells were tested weekly for cytotoxicity against D23 target cells using a microcytotoxicity method and their serum was tested for blocking activity against cytotoxicity by lymph node cells from immunized rats. Tumour growth was suppressed when BCG was in contact with tumour cells but tumours grew unhindered if the BCG was given contralaterally. All rats receiving tumour cells, either alone or mixed with BCG, developed cell-mediated cytotoxicity which remained until termination at 35 days. Rats receiving BCG alone showed slight initial cytotoxicity which disappeared after 7 days. Blocking factors appeared in the serum of rats which developed growing tumours but not in rats whose tumours were suppressed by contact with BCG. Splenectomized rats did not differ markedly from intact rats in the in vitro studies or in vivo. It is concluded that development of cell-mediated immunity and blocking factors depends more upon treatment with tumour cells and the subsequent behaviour of the tumour than upon treatment with BCG per se.


*Br. J. Cancer* (1976) **33**, 584

### EFFECT OF BCG ON CELL-MEDIATED CYTOTOXICITY AND SERUM BLOCKING FACTOR DURING GROWTH OF RAT HEPATOMA

M. J. EMBLETON

*From the Cancer Research Campaign Laboratories, University of Nottingham, Nottingham NG7 2RD, England*

Received 29 December 1975 Accepted 19 February 1976

**Summary.—Inbred rats were injected s.c. with cells of syngeneic hepatoma D23, D23 cells + BCG as a mixed inoculum, mixed inoculum one side and D23 alone contralaterally, or BCG alone. Their blood mononuclear cells were tested weekly for cytotoxicity against D23 target cells using a microcytotoxicity method and their serum was tested for blocking activity against cytotoxicity by lymph node cells from immunized rats.**

**Tumour growth was suppressed when BCG was in contact with tumour cells but tumours grew unhindered if the BCG was given contralaterally. All rats receiving tumour cells, either alone or mixed with BCG, developed cell-mediated cytotoxicity which remained until termination at 35 days. Rats receiving BCG alone showed slight initial cytotoxicity which disappeared after 7 days. Blocking factors appeared in the serum of rats which developed growing tumours but not in rats whose tumours were suppressed by contact with BCG. Splenectomized rats did not differ markedly from intact rats in the *in vitro* studies or *in vivo*.**

**It is concluded that development of cell-mediated immunity and blocking factors depends more upon treatment with tumour cells and the subsequent behaviour of the tumour than upon treatment with BCG *per se*.**

It is now well established that Bacillus Calmette Guérin (BCG) has marked anti-tumour properties, as demonstrated by studies with a wide variety of experimental tumours (Baldwin and Pimm, 1971, 1974; Bartlett, Zbar and Rapp, 1972; Chassoux and Salomon, 1975; Moore, Lawrence and Nisbet, 1975; Simmons, Rios and Kersey, 1972). It has been recognized that close contact between tumour cells and BCG often produces better tumour suppression than administration of the BCG at a site distant from the tumour cells, and this effect has been attributed largely to the host response to BCG itself rather than the tumour cells (Bartlett *et al.*, 1972; Moore *et al.*, 1975). However, there is no doubt that an immune response to tumour cells also develops as a result of BCG-induced growth suppression, since rats which have rejected immunogenic tumours as a result of BCG treatment are resistant to further challenge with the same tumour (Baldwin and Pimm, 1973).

Analysis of the tumour-immune response in BCG-treated animals can most conveniently be carried out by means of *in vitro* tests. Bansal and Sjögren (1973) have shown that a polyoma-virus-induced rat tumour could be inhibited if BCG was administered at the time of tumour grafting, and that treated animals developed an increased cell-mediated immunity to the tumour demonstrable by a microcytotoxicity test. If, however, BCG was given after the appearance of a palpable tumour nodule, tumour growth was accelerated. This was accompanied by an increase in serum factors which "blocked" cell-mediated immunity, but there was no increase in direct cell-mediated immunity.

The following report is a study of the development of cell-mediated cytotoxicity and humoral blocking or inhibitory factors in rats treated with cells of an aminoazo-dye-induced hepatoma (D23) and BCG. This tumour can be suppressed by admixture of viable cells with BCG (Baldwin and Pimm, 1973) and its immunological behaviour has been well studied by *in vitro* cytotoxicity methods (Baldwin, Embleton and Robins, 1973; Baldwin, Price and Robins, 1973). It was thus possible to set up experimental groups with a fairly predictable course of tumour development and to evaluate the immune response of individual rats over a period of time in order to attempt correlations between the host anti-tumour response and the behaviour of the tumour in response to BCG treatment.

### MATERIALS AND METHODS

*Animals.*—Inbred Wistar rats were used. These rats accept skin grafts between individuals of the same sex and strain.

*Tumours.*—The tumour mainly used in these studies was a moderately immunogenic Wistar rat hepatoma, D23, originally induced by oral administration of 4-dimethylamino-azobenzene and maintained by serial s.c. transplantation in syngeneic male rats. Cell suspensions for use *in vivo* were prepared by trypsinization of solid tumour tissue, suspensions consisting of more than 90% viable cells being obtained.

A cell line was maintained in monolayer culture in glass bottles using Eagle's Minimum Essential Medium (MEM) supplemented with calf serum (10%) penicillin (100 iu/ml) and streptomycin (200 $\mu$g/ml). This line was initiated from transplanted tumour tissue and was used as a source of target cells for *in vitro* microcytotoxicity assays. The cultured cells were harvested with 0·25% trypsin and always consisted of 100% viable single cell suspensions. For specificity tests, monolayer cultures of two 3-methylcholanthrene-induced sarcomas, Mc7 and Mc57, were also used.

*BCG.*—Dried percutaneous BCG vaccine was supplied by Glaxo Laboratories Ltd., Greenford, Middlesex. This was reconstituted with distilled water (0·3 ml per ampoule) immediately before use.

*Immunization.*—Groups of animals were treated by one of the following protocols:

(a) $5 \times 10^4$ viable D23 cells given s.c. in the right flank.
(b) 1 mg BCG s.c. in the left flank.
(c) $5 \times 10^4$ viable D23 cells + 1 mg BCG mixed and given s.c. in the left flank.
(d) $5 \times 10^4$ D23 cells + 1 mg BCG mixed in the left flank and $5 \times 10^4$ D23 cells alone in the right flank.
(e) $5 \times 10^4$ D23 cells + 1 mg BCG mixed in the left flank and $5 \times 10^4$ irradiated (15,000 R) D23 cells in the right flank.
(f) Mock splenectomy followed by $5 \times 10^4$ viable D23 cells in the right flank.
(g) Splenectomy followed by $5 \times 10^4$ viable D23 cells in the right flank.
(h) Splenectomy followed by $5 \times 10^4$ D23 cells + 1 mg BCG mixed in the left flank and $5 \times 10^4$ viable D23 cells in the right flank.

Mixtures of D23 cells and BCG were prepared immediately before injection.

*Microcytotoxicity tests.*—Cultured D23 cells were plated at 200/well in Cooke M29 ART Microtitre plates using MEM+10% calf serum. The cells were incubated for between 4 and 24 h to allow them to attach to the bottom of the wells.

Blood mononuclear cells were prepared from individual treated and control rats at weekly intervals until tumour sizes (in groups which developed growing tumours) necessitated termination of the experiments (4–5 weeks after initial treatment). Effector cells from this source were used because this allowed repeated testing of individual rats. Heparinized blood (2 ml) obtained by cardiac puncture was layered on to 2 ml of a mixture of Ficoll (6·35% w/v) and Triosil (13·4% v/v) in a 7 ml disposable plastic bijou. The blood was centrifuged at 400 *g* for 20 min and the supernatant plasma was removed and stored at −20°C for serum blocking factor assay. The mononuclear cell band at the interface between plasma and Ficoll–Triosil was collected and the cells washed twice in MEM before being adjusted to a final concentration of $5 \times 10^5$/ml. Giemsa-stained smears showed the granulocyte contamination to be less than 2%. Medium was removed from the plated D23 target cells and replaced by mononuclear cells at $10^5$/well in 0·2 ml of medium. After 45 min incubation at 37 °C the wells were supplemented with calf serum to a final concentration of 10%. Mononuclear cells from untreated control rats were tested at the same time as those from treated rats and 8 wells were used for each cell preparation. The plates were incubated for 2 days at 37 °C and then gently rinsed with saline to remove all non-adherent cells. The remaining tumour cells were fixed with methanol, stained with Giemsa and counted under $\times 30$ stereoscopic magnification. Percentage cytotoxicity was calculated by the formula:

$$\% \text{ cytotoxicity} = 100 \times \frac{\begin{array}{c}\text{Mean No. of cells}\\ \text{in wells exposed to}\\ \text{control mononuc-}\\ \text{lear cells}\end{array} - \begin{array}{c}\text{Mean No. of cells}\\ \text{in wells exposed to}\\ \text{test mononuclear}\\ \text{cells}\end{array}}{\text{Mean No. of cells in wells exposed to control mononuclear cells}}$$

*Blocking factor assay.*—D23 target cells were plated at 200/well and allowed to attach to the bottom of the wells. Lymph node cells (LNC) were prepared from control rats or rats immunized with 15,000 R-irradiated grafts of hepatoma D23, as previously described (Baldwin *et al.*, 1973*a*). These lymph node cells have been shown to react specifically against the immunizing tumour (Baldwin *et al.*, 1973*a*; Zöller, Dickinson and Embleton, 1975), and were therefore used to provide an effector cell preparation as near standard as possible. Blood plasma was isolated from the supernatant during the preparation of mononuclear cells from heparinized blood. Plasma from treated or control rats was diluted 1/5 in MEM and 0·05 ml aliquots were added to the target cells after removal of the supernatant medium. The cells were incubated for 45 min at 37 °C and $2 \times 10^5$ LNC were added in 0·2 ml MEM without removing the plasma. After a further 45 min calf serum was added to a concentration of 10% and the plates were incubated for 2 days at 37 °C. Percentage blocking was calculated by comparing the % cytotoxicity of immune LNC in the presence of test plasma with % cytotoxicity in the presence of control plasma as described previously (Baldwin *et al.*, 1973*a*).

### RESULTS

Growth of D23 hepatoma cells was suppressed in all rats receiving a mixed inoculum of $5 \times 10^4$ tumour cells and 1 mg BCG, while the same dose of tumour cells alone grew progressively (Table I, Fig. 1). In animals receiving a mixed inoculum of tumour cells and BCG on one side and a simultaneous contralateral inoculum of D23 cells only, the contralateral inoculum grew more slowly than in rats not treated with BCG (Fig. 1), but complete suppression was only observed in 1/11 animals (Table I).

FIG. 1.—Effect of BCG on growth of rat hepatoma D23 locally and at a distant site. BCG was administered throughout at a dose of 1 mg/rat and D23 at $5 \times 10^4$ cells per inoculum. RHS indicates the inoculum was given s.c. on the right flank and LHS indicates the left flank. Vertical bars indicate standard errors of mean values for between 3 and 11 rats (see Table 1).

Cell-mediated cytotoxicity against D23 cells by blood mononuclear cells was observed on repeated occasions in all animals treated with tumour cells, whether or not the cells were suppressed by admixture with BCG (Table I). Rats developing growing tumours had cytotoxic cells up to 35 days after inoculation, at which time the size of the tumours necessitated termination of the experiments (Fig. 2). In rats whose tumour was completely suppressed by admixture with BCG, however, cytotoxicity gradually decreased after 7 days and levels detected after 28 days were insignificant (Fig. 2). Another group were given cells

TABLE I.—*Influence of BCG on Growth of Rat Hepatoma D23 and the Development of Tumour-directed Cell-mediated Cytotoxicity and Serum Blocking Factor*

| | Number of rats positive† for | | |
|---|---|---|---|
| Treatment* | Tumour growth | Cell-mediated cytotoxicity | Serum blocking |
| D23 cells only | 8/8 | 8/8 | 8/8 |
| BCG only | 0/4 | 0/4 | 0/4 |
| D23 cells + BCG mixed | 0/8 | 8/8 | 1/8 |
| D23 cells + BCG mixed, and contralateral D23 cells | 10/11 | 11/11 | 10/11 |
| D23 cells + BCG mixed, and contralateral irradiated‡ D23 cells | 0/4 | 4/4 | 1/4 |
| Mock splenectomy and D23 cells | 3/3 | 3/3 | 3/3 |
| Splenectomy and D23 cells | 3/3 | 3/3 | 3/3 |
| Splenectomy, D23 cells + BCG mixed, and contralateral D23 cells | 4/4 | 4/4 | 4/4 |

* D23 cells and BCG were injected s.c.
† Positive on at least two occasions throughout the time course.
‡ 15,000 R $^{60}$Co $\gamma$-irradiation.

FIG. 2.—Cell-mediated cytotoxicity against hepatoma D23 cells by rats treated with D23 cells and/or BCG.

FIG. 3.—Blocking of cell-mediated cytotoxicity against hepatoma D23 cells by plasma from rats treated with D23 cells and/or BCG.

inactivated by $^{60}$Co-irradiation (15,000 R) and these showed a similar decline of cytotoxicity after 7 days. Rats injected only with 1 mg BCG showed a low level of cytotoxicity at 7 days, but this was transient and no significant cytotoxicity was detectable at 14 days or later (Fig. 2).

Serum blocking activity was also demonstrable in all rats developing growing tumours (Table I), in agreement with previous studies on hosts bearing hepatoma D23 (Baldwin *et al.*, 1973*a*). This was detectable at 7 days and continued to be present up to 35 days, fairly constant levels giving around 80% to 90% blocking being observed throughout the time course. Rats not developing growing tumours did not have significant blocking activity at any stage, although a small insignificant degree of blocking appeared during the first 2 weeks (Fig. 3). These animals included those whose tumour was inhibited by contact with BCG, rats treated with BCG alone and the single rat whose contralateral tumour inoculum was suppressed by an injection of tumour cells + BCG (Table I). Of 4 rats whose contralateral tumour was deliberately prevented from growing by irradiation, blocking activity was observed in only one (Table I).

*Splenectomized rats*

Tumours resulting from an injection of 5 × $10^4$ D23 cells developed in splenectomized rats at a slightly faster rate than in intact or sham-operated controls, but in animals simultaneously given a mixed inoculum of BCG and tumour cells on the opposite flank the rate of growth was retarded (Fig. 4), as in intact rats (Fig. 1). Thus splenectomy had no influence on the weak immunotherapeutic effect of the mixed inoculum on the contralateral tumour.

Cell-mediated cytotoxicity was not impaired in splenectomized rats, being about 50% at Day 7 and continuing at about the same or a slightly increased level up to 28 days (Fig. 5). Splenectomized animals which had been given an inoculum of BCG and D23 cells in addition to contralateral tumour cells also had a moderately high level of cytotoxic mononuclear cells at 7 days, but this diminished to a fairly low level at 21–28 days (Fig. 5).

Serum blocking activity was demonstrable in all groups, but it developed more slowly than in intact rats and did not reach their value until 14 days (Fig. 6) in splenectomized rats. Serum blocking factor remained detectable in all animals up to 28 days, although there was some decrease in the group treated with mixed BCG and tumour cell vaccine. The reduction of blocking activity in this group appeared later than the reduction of cytotoxicity (Fig. 5) which was also observed.

FIG. 6.—Blocking of cell-mediated cytotoxicity against hepatoma D23 by plasma from splenectomized rats treated with D23 cells + BCG.

FIG. 4.—Effect of BCG-tumour-cell vaccine treatment on growth of hepatoma D23 in splenectomized rats.

FIG. 5.—Cell-mediated cytotoxicity against D23 cells by splenectomized rats treated with D23 cells + BCG.

*Specificity tests*

Previous studies have indicated that cytotoxicity against rat hepatoma cells by lymph node cells from immunized or tumour-bearing animals is tumour-specific, with little cross-reaction between tumours with antigens shown to be distinct by transplantation tests (Baldwin *et al.*, 1973*a*). The specificity of action of mononuclear cells prepared from heparinized blood of animals bearing tumours developing from trypsinized cell suspensions is shown in Table II, which lists cross-tests between hepatoma D23 and two sarcomata, Mc7 and Mc57. These three tumours have distinct tumour-rejection antigens which do not cross-react *in vivo*. The data show that blood mononuclear cells from rats bearing hepatoma D23 are cytotoxic for D23 cells but have only an insignificantly small effect on sarcoma cells compared with control mononuclear cells. Cells from sarcoma-bearing rats were reactive

TABLE II.—*Specificity of Cytotoxicity by Blood Mononuclear Cells from Tumour-bearing Rats*

| Target cells | Mononuclear* cell donor | No. of surviving† target cells | Percentage cytotoxicity |
|---|---|---|---|
| D23 | Normal control | 36±2 | — |
| | D23 tumour bearer, 21 days | 5±1 | 86 ($P<0\cdot001$) |
| | Mc7 tumour bearer, 21 days | 30±2 | 17 |
| | Mc57 tumour bearer, 21 days | 31±2 | 14 |
| Mc7 | Normal control | 57±3 | — |
| | D23 tumour bearer, 21 days | 53±2 | 6 |
| | Mc7 tumour bearer, 21 days | 15±1 | 73 ($P<0\cdot001$) |
| Mc57 | Normal control | 43±2 | — |
| | D23 tumour bearer, 21 days | 37±2 | 14 |
| | Mc7 tumour bearer, 21 days | 38±3 | 12 |
| | Mc57 tumour bearer, 21 days | 12±2 | 71 ($P<0\cdot001$) |

* Tumour-bearing donors were injected s.c. with trypsinized tumour cells which were allowed to grow for 21 days.

† Mean number of cells per well ± standard error.

against their respective tumour, but did not significantly alter the survival of other target cells, including D23. These results were obtained with rats bearing tumours for 21 days, which is beyond the point at which the slight non-specific activity was observed in BCG-treated rats (Fig. 2), and show that the cell-mediated cytotoxicity seen against D23 throughout the later part of the time course was tumour-specific.

Serum-mediated blocking of cell-mediated immunity has also previously been reported to be specific for given tumours, using serum prepared from clotted blood of tumour-bearing animals (Baldwin *et al.*, 1973*a*, *b*). In the present study, however, plasma was isolated from heparinized blood in order to assay blocking activity in the same samples as were used for effector cell preparation. In order to test whether heparin produced non-specific effects, a further series of cross-tests were carried out as shown in Table III. Lymph node cells from immune donors were used as effector cells in the majority of the tests because these were known to be tumour-specific in activity (Baldwin *et al.*, 1973*a*). In each test the base-line was percentage cytotoxicity in the presence of normal rat plasma. Against this baseline, cytotoxicity against D23 by immune lymph node cells was diminished by treatment with D23 tumour-bearer plasma but not by plasma from rats bearing either sarcoma Mc7 or Mc57. Similarly, D23 tumour-bearer plasma did not block cytotoxicity against Mc7 or Mc57 target cells by specifically immune lymph node cells. Thus, blocking activity detectable in tumour-bearer plasma had tumour-related specificity which appeared to be unaffected by the use of heparin.

Included in Table III are tests of plasma blocking activity against D23 tumour-bearer blood mononuclear cells. Cytotoxicity against D23 mediated by these cells was blocked by D23 tumour-bearer plasma, but not by Mc7 tumour-bearer plasma. This indicates that the types of effector cells used to monitor cytotoxicity in Table I (Figs. 2 and 5) were also subject to the blocking effects of humoral factors in tumour-bearing animals.

### DISCUSSION

The growth of hepatoma D23 could be suppressed by admixture of tumour cells with BCG as previously reported (Baldwin and Pimm, 1973). Growth of

TABLE III.—*Specificity of Blocking of Cell-mediated Cytotoxicity by Plasma from Tumour-bearing Rats*

| Target cells | Effector* cells | Plasma† donor | % Cytotoxicity | % Blocking |
|---|---|---|---|---|
| D23 | D23-immune LNC | Normal control | 50 ($P<0\cdot001$) | — |
| | | D23 tumour bearer | 6 | 88 ($P<0\cdot01$) |
| | | Mc7 tumour bearer | 44 ($P<0\cdot01$) | 12 |
| | | Mc57 tumour bearer | 46 ($P<0\cdot01$) | 8 |
| D23 | D23 tumour-bearer BM | Normal control | 52 ($P<0\cdot01$) | — |
| | | D23 tumour bearer | 10 | 81 ($P<0\cdot01$) |
| | | Mc7 tumour bearer | 48 ($P<0\cdot01$) | 8 |
| Mc7 | Mc7-immune LNC | Normal control | 51 ($P<0\cdot001$) | — |
| | | D23 tumour bearer | 51 ($P<0\cdot001$) | 0 |
| | | Mc7 tumour bearer | −6 | 100 ($P<0\cdot01$) |
| Mc57 | Mc57-immune LNC | Normal control | 55 ($P<0\cdot001$) | — |
| | | D23 tumour bearei | 54 ($P<0\cdot001$) | 2 |
| | | Mc57 tumour bearer | 6 | 89 ($P<0\cdot01$) |

* Immune LNC = lymph node cells from tumour-immune rats. Tumour bearer BM = blood mononuclear cells from rats bearing tumours growing from trypsinized D23 cells. Similar effector cells from normal rats were used as controls.

† Plasma was isolated from heparinized blood of rats bearing tumours growing for 21 days from s.c. injected trypsinized cells.

a contralateral inoculum of tumour cells alone given simultaneously, however, was not completely suppressed but showed retardation compared with tumour growth in untreated control rats. Similar retardation of tumour growth by a distant inoculation of D23 cells mixed with BCG occurred in splenectomized rats, although the overall rate of tumour growth was slightly accelerated in these animals compared with equivalent groups whose spleen was intact. Using strongly immunogenic polyoma-virus-induced rat tumours, Bansal and Sjögren (1972, 1973) have shown that splenectomy in their system can lead to retardation or regression of tumours if combined with immune serum or BCG treatment.

Cell-mediated cytotoxicity developed in all rats treated with D23 tumour cells, whether or not these grew progressively or were suppressed by BCG treatment or $\gamma$-irradiation. Animals with growing tumours exhibited strong cytotoxicity right up to the time of termination (5 weeks), while those with suppressed tumours showed a decline in cytotoxicity after 14 days although cytotoxic cells were still detectable up to 28 days after inoculation. The animals with BCG-suppressed tumours at no time showed stronger reactivity than those given tumour cells alone, contrary to the findings of Bansal and Sjögren (1973) who reported that cell-mediated cytotoxicity to polyoma tumours was increased in BCG-treated rats.

Animals treated with BCG alone showed a slight transient cell-mediated cytotoxicity at 7 days, which was not evident thereafter. This could have been due to the presence of activated monocytes in the mononuclear cell suspensions in the early stages after treatment, since it has been shown that BCG-primed macrophages are cytotoxic for tumour cells (Evans and Alexander, 1972). No attempt had been made to separate monocytes from lymphocytes in the present studies, since it was felt that either cell type might act as an effector cell in the BCG immunotherapy model employed.

Serum factors blocking cell-mediated cytotoxicity were assayed with the test serum present during the whole period of incubation of effector cells with target cells, rather than by pretreatment of effector cells or target cells as in previous studies (Baldwin *et al.*, 1973*a*, *b*). Under these conditions, significant blocking was found in all animals which developed growing tumours, including both intact and splenectomized animals. Serum from rats bearing D23 tumour has previously been shown to contain factors which can block cytotoxicity when applied to the target cells (Baldwin *et al.*, 1973*a*; Bowen, Robins and Baldwin, 1975) or to the effector cells (Baldwin *et al.*, 1973*b*; Bowen *et al.*, 1975). In both situations the blocking effect has been attributed to complexes of tumour antigen and tumour-specific antibody, and inhibition at the effector cell level can also be accomplished by treatment with tumour antigen alone. Bansal and Sjögren (1972, 1973) have interpreted the therapeutic effects of splenectomy in their polyoma rat tumour system on the basis of loss of blocking activity owing to the lack of circulating complexes resulting from reduced antibody-forming capability. This was supported by *in vitro* tests showing a loss of blocking activity in serum when tested by pretreatment of target cells. In the situation where serum is present throughout the incubation, however, it is conceivable that the blocking effect is due to inhibition of effector cells by soluble antigen shed by the tumour in the case of splenectomized animals, and either by antigen or immune complexes in intact animals. The fact that blocking activity was detected later in splenectomized rats than in rats with intact spleens lends support to the idea that different mechanisms were operating, although the end effect was the same in both situations.

In view of the time lag in blocking factor detection, there was no correlation between blocking measured *in vitro* and the slightly accelerated development of tumours in the splenectomized groups. Neither can the growth rate be explained on the basis of reduced cell-mediated immunity, since cell-mediated cytotoxicity was equally high in intact and splenectomized rats. In contrast to tumour-bearing rats, no blocking factor was detectable in rats which failed to develop tumours. This was not attributable to BCG treatment, since BCG-treated rats which grew contralateral tumours had considerable blocking activity, but was correlated only with the lack of growing tumours.

These studies were undertaken in order to ascertain whether any differences could be found in animals given BCG immunotherapy and/or splenectomy compared with normal tumour-bearing animals, correlated with tumour growth. This is an important concept in relation to current cancer immunotherapy trials in man, where attempts are being made to correlate the clinical course of the disease with *in vitro* measurements of tumour-directed immunity, as exemplified by trials with malignant melanoma (Berkelhammer *et al.*, 1975; Currie, 1973; Currie and McElwain, 1975). In the rat hepatoma system, where only weak immunotherapeutic effects were obtained, there was a strong correlation between tumour growth and serum blocking activity, but no correlation between BCG treatment and blocking factor development. Cell-mediated cytotoxicity was comparable in all groups of tumour-treated rats whether they received immunotherapy, splenectomy or no other treatment. The development of cytotoxicity was dependent only upon treatment with tumour D23, and the development of blocking activity was dependent only upon whether the tumour was suppressed or allowed to grow. BCG was only effective in so far that it produced suppression of tumour cells in contact with it and splenectomy had little effect on any of the immune parameters measured, so it must be concluded that neither of these treatments affected the *in vitro* measurements *per se*.

The author wishes to thank Mrs B. A. Jones for skilled technical assistance. This work was supported by a block grant from the Cancer Research Campaign and BCG vaccine was kindly supplied by Glaxo Research Ltd.

### REFERENCES

BALDWIN, R. W., EMBLETON, M. J. & ROBINS, R. A. (1973*a*) Cellular and Humoral Immunity to Rat Hepatoma—Specific Antigens Correlated with Tumour Status. *Int. J. Cancer*, **11**, 1.

BALDWIN, R. W. & PIMM, M. V. (1971) Influence of BCG Infection on Growth of 3-methylcholanthrene-induced Rat Sarcomas. *Eur. J. clin. biol. Res.*, **16**, 875.

BALDWIN, R. W. & PIMM, M. V. (1973) Immunotherapy of Rat Tumours of Defined Immunogenicity. *Natl Cancer Inst. Monog.*, **39**, 11.

BALDWIN, R. W. & PIMM, M. V. (1974) BCG Suppression of Pulmonary Metastases from Primary Rat Hepatomas. *Br. J. Cancer*, **30**, 473.

BALDWIN, R. W., PRICE, M. R. & ROBINS, R. A. (1973*b*). Inhibition of Hepatoma Immune Lymph Node Cell Cytotoxicity by Tumour Bearer Serum and Solubilised Hepatoma Antigen. *Int. J. Cancer*, **11**, 527.

BANSAL, S. C. & SJÖGREN, H. O. (1972) Counteraction of the Blocking of Cell-Mediated Tumour Immunity by Inoculation of Unblocking Sera and Splenectomy: Immunotherapeutic Effects on Primary Polyoma Tumours in Rats. *Int. J. Cancer*, **9**, 490.

BANSAL, S. C. & SJÖGREN, H. O. (1973) Effects of BCG on Various Facets of the Immune Response against Polyoma Tumours in Rats. *Int. J. Cancer*, **11**, 162.

BARTLETT, G. L., ZBAR, B. & RAPP, H. J. (1972) Suppression of Murine Tumor Growth by Immune Reaction to the Bacillus Calmette-Guérin Strain of *Mycobacterium bovis*. *J. natn. Cancer Inst.*, **48**, 245.

BERKELHAMMER, J., MASTRANGELO, M. J., LAUCIUS, J. F., BODURTHA, A. J. & PREHN, R. T. (1975) Sequential *In Vitro* Reactivity of Lymphocytes from Melanoma Patients receiving Immunotherapy Compared with the Reactivity of Lymphocytes from Healthy Donors. *Int. J. Cancer*, **16**, 571.

BOWEN, J. G., ROBINS, R. A. & BALDWIN, R. W. (1975) Serum Factors Modifying Cell-Mediated Immunity to Rat Hepatoma D23 Correlated with Tumour Growth. *Int. J. Cancer*, **15**, 640.

CHASSOUX, D. & SALOMON, J.-C. (1975) Therapeutic Effect of Intratumoral Injection of BCG and Other Substances in Rats and Mice. *Int. J. Cancer*, **16**, 515.

CURRIE, G. A. (1973) Effect of Active Immunisation with Irradiated Tumour Cells on Specific Serum Inhibitors of Cell-Mediated Immunity in Patients with Disseminated Cancer. *Br. J. Cancer*, **28**, 25.

CURRIE, G. A. & MCELWAIN, T. J. (1975) Active Immunotherapy as an Adjuvant to Chemotherapy in the Treatment of Disseminated Malignant Melanoma; A Pilot Study. *Br. J. Cancer*, **31**, 143.

EVANS, R. & ALEXANDER, P. (1972) Mechanism of Immunologically Specific Killing of Tumour Cells by Macrophages. *Nature* (*Lond.*), **236**, 168.

MOORE, M., LAWRENCE, N. & NISBET, N. W. (1975) Tumour Inhibition Mediated by BCG in Immunosuppressed Rats. *Int. J. Cancer*, **15**, 897.

SIMMONS, R. L., RIOS, A. & KERSEY, J. M. (1972) Regression of Spontaneous Mammary Carcinoma Using Direct Injections of Neuraminidase and BCG. *J. surg. Res.*, **12**, 57.

ZÖLLER, M., DICKINSON, A. M. & EMBLETON, M. J. (1975) Cell-Mediated Immune Reactions against Rat Tumour Cells Detected by *In Vitro* Microcytotoxicity Assays. *Br. J. Cancer*, **32**, 240.